# Molecular aspects of heat stress sensing in land plants

**DOI:** 10.1111/tpj.70069

**Published:** 2025-03-14

**Authors:** Cristiane Paula Gomes Calixto

**Affiliations:** ^1^ Department of Botany, Institute of Biosciences University of São Paulo São Paulo Brazil

**Keywords:** Oryza, Arabidopsis, abiotic stress, heat, high temperature, ROS, DNA damage, RNA binding proteins, molecular chaperones, membrane biology

## Abstract

Heat stress impacts all aspects of life, from evolution to global food security. Therefore, it becomes essential to understand how plants respond to heat stress, especially in the context of climate change. The heat stress response (HSR) involves three main components: sensing, signal transduction, and cellular reprogramming. Here, I focus on the heat stress sensing component. How can cells detect heat stress if it is not a signalling particle? To answer this question, I have looked at the molecular definition of heat stress. It can be defined as any particular rise in the optimum growth temperature that leads to higher‐than‐normal levels of reactive molecular species and macromolecular damage to biological membranes, proteins, and nucleic acid polymers (DNA and RNA). It is precisely these stress‐specific alterations that are detected by heat stress sensors, upon which they would immediately trigger the appropriate level of the HSR. In addition, the work towards thermotolerance is complemented by a second type of response, here called the cellular homeostasis response (CHR). Upon mild and extreme temperature changes, the CHR is triggered by plant thermosensors, which are responsible for monitoring temperature information. Heat stress sensors and thermosensors are distinct types of molecules, each with unique modes of activation and functions. While many recent reviews provide a comprehensive overview of plant thermosensors, there remains a notable gap in the review literature regarding an in‐depth analysis of plant heat stress sensors. Here, I attempt to summarise our current knowledge of the cellular sensors involved in triggering the plant HSR.

## Significance Statement

Understanding how plants sense heat stress is crucial for improving crop resilience and protecting biodiversity, especially in the face of climate change. Here, I review the cellular sensors involved in detecting heat‐stress‐related stimuli in land plants, upon which they trigger the HSR and help drive thermotolerance.

## THE MOLECULAR DEFINITION OF HEAT STRESS

**Figure 1:**
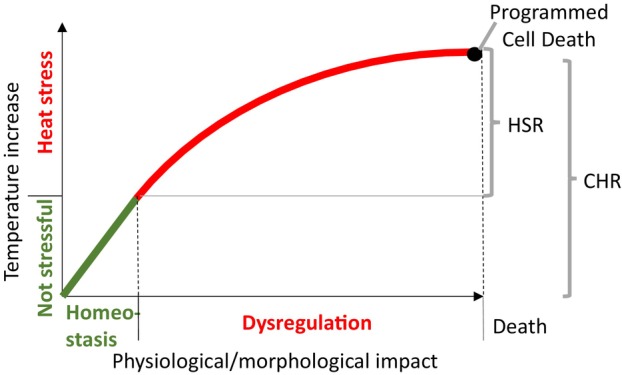
Relationship between temperature increase and its physiological/morphological impact on a living system. Here, the term ‘temperature increase’ refers to the intensity and/or duration of an environmental temperature increase from the optimum growth range (not stressful) of a given living system. The cellular homeostasis response (CHR) mechanism permanently works towards maintaining cellular homeostasis (green). In heat stress conditions (red), the system is dysregulated and the work towards thermotolerance is complemented by the heat stress response (HSR). The cellular response to extreme heat stress can end with the programmed cell death mechanism. Adapted from Kültz (2020).

Temperature stress is one of the strongest forces driving evolution and limiting species distribution around the globe (Bennett et al., 2021; Normand et al., 2009; Willi & Van Buskirk, 2022). Moreover, current and predicted human‐driven temperature anomalies, particularly rising temperatures, pose one of the biggest threats to biodiversity and global food security (Gao et al., 2020; Lesk et al., 2016; Rezaei et al., 2023; Zhao et al., 2016). To develop better mitigation strategies in this scenario, it becomes essential to understand how organisms perceive and respond to heat stress. However, studying it is not straightforward. Complex biological systems, such as land plants, have emergent properties that considerably increase the complexity of stress effects and responses (Bertolli et al., 2014; Street et al., 2011). Even defining biological stress can be quite challenging – it can vary according to the type of stress being studied and the biological question being investigated (Mosa et al., 2017; Schulte et al., 2014). When looking at the molecular level, heat stress is often defined as any particular rise in the optimum growth temperature that pushes the physiological status of a cell beyond its homeostatic range, leading to a system dysregulation (Figure 1). This definition is used hereafter. A dysregulated system generally exhibits higher‐than‐normal levels of reactive molecular species and macromolecular damage – both are ubiquitous effects of most stressful conditions, including heat stress (Kültz, 2005). These effects likely occurred frequently, with lasting and/or intense impacts early in the history of life, providing crucial conditions in which a general mechanism of stress response system could be shaped by natural selection. This general mechanism of stress response is known as the cellular stress response and it is conserved, all living systems have it, playing a crucial role in the cells' defence against virtually all stressors (Dietz & Vogelsang, 2024; Kültz, 2005). Having said that, some stress‐specificity can also be observed in this response, which is further explored in this Focused Review with an emphasis on heat stress. Additionally, heat stress can compromise other stress responses, such as plant defences against biotic stress, revealing that plant responses to a combination of heat and other stresses are unique (Kan et al., 2023; Zandalinas et al., 2021). In summary, stressful heat conditions can dysregulate a living system to a point at which it will lack the energy, time and/or the complete set of resources to maintain its metabolic activities at the highest levels, which can result in reductions in performance/fitness (Schulte et al., 2014).

## HEAT STRESS ADAPTATION STRATEGIES

The evolutionary development of strategies, mechanisms and elements involved in plant adaptation to high‐temperature stress is highly complex. From an evolutionary perspective, the costs of increased heat stress adaptation do not always outweigh the benefits. For example, maintaining excess capacities — such as the constant expression of heat shock proteins when there is limited to no exposure to heat stress — can be negatively selected, likely due to the wasted energy and resources (Diamond, 2002). This complexity extends to the molecular level, where the function of large biomolecules is intrinsically dependent on the fine balance between their molecular stability and structural flexibility, making them easily vulnerable to heat stress (Somero, 2020). As a result, plants have a restricted limit of high temperatures within which they can thrive. Phylogenetic analyses further support the existence of a stronger physiological boundary limiting species adaptation to heat when compared with cold (Bennett et al., 2021). Therefore, heat stress adaptation is continuously under selective pressure and varies widely among organisms, ultimately shaping the different strategies that have allowed life to flourish across different ecosystems on Earth.

Land plants have been particularly successful in colonising the arduous terrestrial environments and they transformed our planet in the process (Donoghue et al., 2021). Their adaptation to heat stress can be categorised into three primary strategies: escape, resistance and tolerance. The escape strategy enables plants to skip stress altogether by altering their seasonal phenology, growth and development so they only occur during a favourable period (Willi & Van Buskirk, 2022). Stress resistance involves not only an innate morpho‐physiological state but also short‐ or long‐term modifications that enable the plant to keep its internal conditions within the optimal range, even under external stressful conditions. For example, leaf temperatures can go above or below air temperatures through mechanisms, such as transpiration, leaf movements and controlled trichome and canopy densities (Helliker & Richter, 2008; John‐Bejai et al., 2013). Lastly, mechanisms of heat stress tolerance, known as thermotolerance, serve three main functions: (i) aiding plants in maintaining and adjusting certain metabolic activities despite heat stress‐induced system dysregulation, (ii) enhancing cellular plasticity to maintain some level of functionality under elevated temperature conditions, and (iii) promoting acclimation to heat. It is noteworthy that thermotolerance varies not only between species but also within an individual plant, depending on factors, such as the time of day, season, tissue and developmental stage (Dickinson et al., 2018; Grossman, 2023; Huang et al., 2012). Moreover, thermotolerance can be acquired through preexposure to a mild environmental change, known as priming (Bäurle, 2016), a process influenced by the ontogenetic age of the plant or tissue (van Buer et al., 2019). Among these three adaptation strategies, thermotolerance is perhaps the most dependent on the perception of heat stress information, as this ability is essential for activating the HSR. In other words, without accurate heat stress perception, the plant would fail to activate the HSR, hindering the full development of thermotolerance.

## THE HEAT STRESS RESPONSE (HSR) AT THE MOLECULAR LEVEL

**Figure 2:**
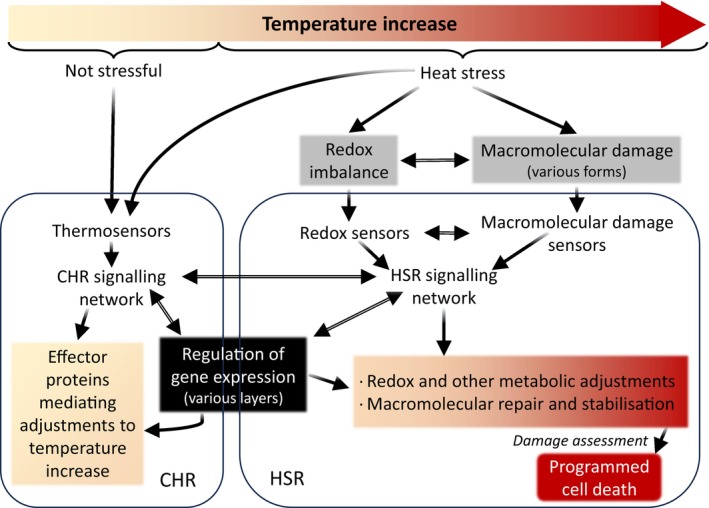
Interconnected molecular responses to temperature increases in plants. The term ‘temperature increase’ refers to both the intensity and/or duration of an environmental rise in temperature. Upon heat stress, the HSR repress growth and helps restore macromolecular integrity and redox balance, while the CHR works in parallel to re‐establish homeostasis/allostasis. Genes encoding thermosensors, redox sensors and macromolecular damage sensors are also regulated in response to temperature increases; however, for clarity, this regulation is not depicted in the figure. Adapted from Kültz (2005).

Numerous genetic, biochemical and molecular studies have identified many factors that compose the HSR in plants. Similarly to most signal transduction networks, plants respond to heat stress through three key components: (a) sensors that detect heat stress; (b) transducers that receive, amplify and coordinate the flow of information initially given by the sensors; and (c) effectors that modify cellular activities in response to the signals passed on by the transducers. Heat stress elicits several layers of cellular responses, including transcriptional, post‐transcriptional, translational and post‐translational modifications (Kan et al., 2023). The main functions of the HSR are to repress growth, modulate key energy metabolism pathways, restore redox balance, and reduce stress‐induced damage. In this process, it helps the cellular homeostasis/allostasis response (CHR) in its attempt at reinstating homeostasis/allostasis during stress, thereby conferring thermotolerance (Kültz, 2020) (Figure 1). Although the HSR and the CHR are linked and contain common elements, they are different types of responses, with many sensing‐ and signalling‐specific pathways (Figure 2) (Kan et al., 2023; Kültz, 2005). Organisms have evolved more complex stress responses from the general cellular stress response (Kültz, 2020), such as the programmed cell death (PCD), which removes cells with stress‐induced, life‐threatening damage. To effectively determine the most appropriate response to varying levels of heat stress, cells must accurately assess the severity of the stress, a task performed by heat stress sensors, which will be the primary focus of this review. For more information on transducers and effectors of the HSR please refer to recent reviews (Kan et al., 2023; Sato et al., 2024).

## HEAT STRESS SENSING

A typical signal transduction process initiates with a signalling particle being perceived by sensors. However, this is not the case for heat stress. To begin with, heat is not a particle; rather, it is a process of energy transfer between bodies due to a temperature difference (Doige & Day, 2012). When this transfer occurs from the environment to the plant, the temperature of cellular components increases, consequently changing their physical and chemical properties. During mild temperature increases, within the optimum range, plants can maintain cellular homeostasis/allostasis through a series of pathways and adjustments. These involve key players such as thermosensors, responsible for monitoring and transferring temperature information to the CHR, calcium (Ca^2+^) signalling proteins, and specific effector proteins that mediate warm‐specific changes, such as the thermomorphogenesis (Kerbler & Wigge, 2023). However, if the temperature increase exceeds the optimum range, many cellular systems become impaired and dysregulated, resulting in particular redox imbalances and certain types of macromolecular deformation/damage, namely on lipid membrane, protein, and nucleic acids. It is precisely these stress‐specific alterations that are detected by stress sensors, upon which they would immediately promote and maintain the HSR (Kültz, 2005). Therefore, heat stress sensors and thermosensors are distinct types of molecules, each with unique modes of activation and functions (Table 1). In fact, these two types of sensors have different origins. Stress sensors, along with the cellular response they trigger, are highly conserved across all kingdoms (Kültz, 2003). This response may have later facilitated the independent evolution of environmental‐specific adaptations, such as temperature sensors, which might explain the reduced conservation of thermosensors across different groups of organisms (Guerin et al., 2024; Kültz, 2003; Zhu et al., 2024). While the recent reviews by Kerbler and Wigge (2023) and Casal et al. (2024), among others, provide a comprehensive overview of plant thermosensors, there remains a notable gap in the review literature regarding an in‐depth analysis of plant heat stress sensors. In this Focused Review, I attempt to summarise our current knowledge of the cellular sensors involved in triggering the plant HSR.

**Table 1:** Main differences between heat stress sensors and thermosensors

Heat stress sensors	Thermosensors
Detect heat‐induced macromolecular damage and/or oxidative stress	Detect mild and/or severe temperature changes
In some cases, alterations in their structural conformation due to heat might also be a crucial aspect of their molecular function	In all cases, temperature‐induced changes in their biochemical properties are essential for their function
Trigger the HSR	Trigger the CHR
Drive thermotolerance	Can drive thermotolerance, thermomorphogenesis, among others
Highly conserved sensory mechanism	Multiple independent evolutionary origins across kingdoms

The identification of cellular sensors involved in detecting heat stress‐related stimuli remains challenging. To aid in this identification, there are a few proposed criteria that sensors should follow (Dietz & Vogelsang, 2024). Given the pervasive nature and broad effects of extreme temperature on all cellular components, these sensors must be able to detect relevant heat stress‐induced alterations and rapidly transmit this information to trigger the downstream HSR in a robust manner. The effectiveness of these detection and transmission abilities is especially dependent on the thermostability and structural flexibility of the sensors at higher temperatures. Two other important factors to consider are the quantitative and qualitative aspects of heat stress sensing: specifically, the number of sensors required to trigger the HSR and their localisation within different cellular compartments (Ang et al., 2024; Dietz & Vogelsang, 2024; Mittler et al., 2022). Currently, there is limited knowledge regarding how the intensity of heat stress is perceived and translated into an optimal level of HSR (Dietz & Vogelsang, 2024). Dietz and Vogelsang (2024) propose two types of signalling contexts in which the HSR is activated, depending on the proportion of sensors involved: gain‐of‐function or loss‐of‐function. In the gain‐of‐function signalling context, the activation of a small proportion of sensors (<25%) is sufficient to trigger the HSR, due to mechanisms like signal amplification and positive feedback. In contrast, the loss‐of‐function signalling context refers to the control of the HSR when most sensors (>90%) of a particular type interact with heat stress‐specific signals. This signalling context is attributed to the redundancy and robustness of these pathways, where some sensors rely on the coordinated action of others to interfere in a given pathway, or where widespread inactivation of repressive sensors (de‐repression) removes inhibition, thereby triggering the HSR.

Overall, heat stress sensors mostly sense heat stress indirectly – they can monitor heat stress‐specific types and degrees of redox imbalance and macromolecular deformation or damage, upon which they trigger the appropriate level of the HSR. To guide readers through the broad range of heat stress sensors, I have prepared the four sections below, each dedicated to a specific type of sensor: sensors of redox imbalance, biological membrane damage sensors, protein damage sensors and DNA/RNA damage sensors. A concise summary of most plant heat stress sensors identified to date is presented in Table 2, which also includes gene IDs from two distantly related diploid species, both among the most highly studied (Marks et al., 2023): the dicotyledonous *Arabidopsis thaliana* and the monocotyledonous *Oryza sativa*.

**Table 2:** Representative list of putative plant heat stress sensors

Types of sensors	Illustrative candidate genes		References
	Examples in Arabidopsis	Potential orthologue in rice^†^	
Sensors of redox imbalance			
MAPKs and/or MAPK repressors	AT1G09000 (ANP1)	LOC_Os08g32600 (PI33)	Kovtun et al. (2000), Liu and He (2017), Kumar et al. (2021), Mo et al. (2021)
Transcription factors and coactivators	AT4G18880 (HSFA4A)	LOC_Os01g54550 (HSFA4A)	Suzuki et al. (2013), Andrási et al. (2020), He et al. (2022)
	AT3G24500 (MBF1c)	LOC_Os06g39240 (MBF1c)	
	AT1G13450 (GT‐1)	LOC_Os04g40930 (RML1)	
Inhibitory proteins	AT1G32230 (RCD1)	LOC_Os10g42710 (TWI1)	Muench et al. (2016), Sato et al. (2024)
Annexins	AT1G35720 (ANN1)	LOC_Os06g11800 (ANN5)	Liao et al. (2017), Saad et al. (2020)
Plant thiol peroxidases	AT3G11630 (2CPA)	LOC_Os02g33450	Liu et al. (2020), Vogelsang and Dietz (2022)
Dehydrogenases	AT3G04120 (GAPC)	LOC_Os02g38920 (GAPC1), LOC_Os04g40950 (GAPC2)	Kim et al. (2020), Jethva et al. (2023)
Biological membrane damage sensors			
Channels	AT5G54250 (CNGC4) AT5G15410 (CNGC2)	LOC_Os01g57370 (CNGC1) LOC_Os03g55100 (CNGC7) LOC_Os05g42250 (CNGC9)	Finka and Goloubinoff (2014), Cui et al. (2020), Jarratt‐Barnham et al. (2021), Kerbler and Wigge (2023)
Phospholipases	AT4G35790 (PLDδ)	LOC_Os09g37100 (PLDdelta1)	Kim et al. (2022), Annum et al. (2023)
Protein damage sensors			
HSPs/molecular chaperones	AT5G28540 (BiP1), AT5G42020 (BiP2), AT1G09080 (BiP3)	LOC_Os02g02410 (BIP1)	Srivastava et al. (2013)
	AT5G02500 (HSP70‐1), AT5G02490 (HSP70‐2)	LOC_Os11g47760	Sugio et al. (2009), Li et al. (2010), Andrási et al. (2020)
	AT5G52640 (HSP90.1) AT5G56030 (HSP90.2) AT5G56010 (HSP81‐3) AT5G56000 (Hsp81.4)	LOC_Os09g30412 (HSP82B) Os09g0482400^‡^ (HSP80.2) LOC_Os09g30418	Andrási et al. (2020)
DNA and RNA damage sensors			
MutS family	AT3G24320 (MSH1)	LOC_Os04g42784	Virdi et al. (2016)
MutL family	AT4G35520 (MLH3)	LOC_Os09g37930 (FSV1)	Phillips et al. (2015)
MRN complex	AT5G54260 (MRE11)	LOC_Os04g54340 (MRE11)	Zhao et al. (2023)
UTR of RNAs			Su et al. (2018)
RNA binding proteins	AT1G20220 (ALBA4) AT1G76010 (ALBA5) AT3G07030 (ALBA6)	LOC_Os09g37006 (ALBA7) LOC_Os03g52490 (ALBA3)	Tong et al. (2022)

^†^ *Oryza sativa* ssp. *japonica* genes identified as the best BLAST hit among its paralogs are shown. This analysis used the Arabidopsis genes as the query against the PLAZA protein database and is based on the Best‐Hits‐and‐Inparalogs family approach, available in the Integrative Orthology Viewer of PLAZA 5.0 (Van Bel et al., 2021). When available, the CGSNL rice gene symbol is shown in brackets.

^‡^ The Rice Annotation Project (RAP) gene ID (Sakai et al., 2013).

### Sensors of redox imbalance

Redox reactions were likely fundamental in the origin of life and have always been part of most biological processes (Bromberg et al., 2022). During these reactions, a small amount of electrons is inevitably lost to available oxygen (O₂•) molecules, generating reactive oxygen species (ROS) in the <50 nM range (Dietz & Vogelsang, 2024; Halliwell, 2006; Raimondi et al., 2020). In plants, ROS occurs in four major forms: singlet oxygen (^1^O₂), superoxide anion (O₂•^−^), hydrogen peroxide (H₂O₂) and hydroxyl radical (•OH) (Waszczak et al., 2018). The chemical reactivity of ROS is higher than that of oxygen (Herb et al., 2021), readily oxidising most cellular molecules – a reaction that often results in the loss of function or alteration of macromolecules, as well as the generation of other reactive molecular species (RMS), such as reactive nitrogen species (RNS). Despite these challenges, cells have been benefitting from redox reactions in oxygen‐rich environments while relying on homeostasis for two main reasons. Selection helped shape not only an antioxidant system that tightly controls the levels and location of RMS but also a repair system that fixes or replaces damaged biomolecules (Herb et al., 2021; Kültz, 2005; Waszczak et al., 2018). Moreover, actively producing and utilising manageable levels of reactive species, a phenomenon referred to as oxidative eustress, can provide benefits in regulating metabolism, physiology, immunity and development (Castro et al., 2021; Corpas et al., 2020; Di Meo et al., 2016; Waszczak et al., 2018).

Being complex biological networks, both antioxidant and prooxidant systems are dynamic and robust, often maintaining a balanced redox status against the ever‐changing environment. However, biological networks are also known to be fragile when faced with unexpected or extreme perturbations (Kwon & Cho, 2008), and the anti/prooxidant systems are no different. Generally, stressful environments can dysregulate these systems, for example, uncoupling metabolic pathways, such as electron transport chains, and affecting the activity of enzymes, such as ROS scavengers. Dysregulation of these systems often leads to increased activity of the prooxidant system and decreased efficiency of the antioxidant system (Kültz, 2005; Wang et al., 2018). Consequently, the balance always tips in favour of a sustained and overwhelming increase in RMS levels, establishing the oxidative distress, hereafter referred to as oxidative stress (Halliwell, 2006; Sies, 2018). Depending on the type of stress, the levels of various RMS can increase in different cellular compartments, generating unique cellular stress‐specific ‘landscapes’ of redox imbalance (Ang et al., 2024; Mittler et al., 2022). Mittler et al. (2022) suggests that plant cells can decode these signatures by employing redox sensors in different cellular compartments, ultimately helping trigger the appropriate cellular stress response.

Heat stress can severely impact RMS metabolism, including their generation and propagation, resulting in altered RMS signatures and gradients. Compartment‐specific redox signatures of heat stress involve chloroplasts and mitochondria, which are two major centres of heat‐induced ROS production (Mittler et al., 2022; Suzuki, 2023; Wang et al., 2018). Interestingly, chloroplast‐generated ROS are involved in retrograde signalling, a process where signals originating in organelles like chloroplasts influence nuclear gene expression (Sun & Guo, 2016). During heat stress, oxidation levels in the nucleus are also considerably elevated (Babbar et al., 2021), playing an important role in the HSR (Liu et al., 2020; Sun & Guo, 2016). Additionally, heat‐induced ROS bursts also occur in the extracellular spaces between cells, followed by a rapid systemic signal propagation mediated by RESPIRATORY BURST OXIDASE HOMOLOGUE D (RBOHD) proteins (Miller et al., 2009; Suzuki et al., 2013). Apoplastic ROS can enter the cytoplasm, preferentially via aquaporin channels (Liu & He, 2017). RMS propagation from the primary sites of heat‐induced ROS production also leads to increased oxidation in the cytosol (Babbar et al., 2021). Besides these heat stress‐related RMS signatures and propagation, the intensity of the oxidative stress is a critical factor in the HSR. For example, Wang et al. (2020) demonstrated a clear heat stress dose‐dependent increase in ROS accumulation in Arabidopsis. Similarly, Locato et al. (2008) observed a fast and transient increase in the endogenous levels of nitric oxide — one of the main RNS — in tobacco following heat stress. These findings suggest that RMS levels are involved in establishing different intensities of the HSR. However, further research is needed to fully identify the heat‐specific types of RMS, their signatures, transport mechanisms, and concentrations, as well as how these aspects integrate with each other and with other signal transduction pathways to determine the optimal level of the HSR.

The precise mechanisms underlying the sensing of heat stress‐induced RMS remain mostly scarce, but they probably involve both direct and indirect effects of the oxidative stress on signalling pathways. The direct effect occurs when RMS oxidise signalling proteins, resulting in post‐translational modifications that either activate them, initiating a gain‐of‐function signalling cascade, or undermine their function, triggering a loss‐of‐function signalling response. For example, stress‐related ROS can activate specific plant mitogen‐activated protein kinases (MAPKs) and/or inactivate MAPK repressors, thereby initiating a signalling cascade that activates cellular stress responses, including the transcriptional activation of heat stress‐related genes (Kovtun et al., 2000; Kumar et al., 2021; Liu & He, 2017; Mo et al., 2021). Under heat‐induced oxidative stress conditions, RMS can covalently modify key transcription factors (TFs) and coactivators, such as the HEAT STRESS TRANSCRIPTION FACTOR A4A (HSFA4A), the MULTIPROTEIN BRIDGING FACTOR 1C (MBF1C), and the trihelix TF GT‐1, driving the transcriptional reprogramming observed in the thermotolerance response (Andrási et al., 2020; Babbar et al., 2021; He et al., 2022; Suzuki et al., 2013). In addition, heat stress‐induced RMS can destabilise inhibitory proteins like heat shock proteins (HSP) and the nuclear‐localised protein RADICAL‐INDUCED CELL DEATH1 (RCD1), resulting in the release of TFs and the activation of the HSR (Muench et al., 2016; Sato et al., 2024).

Heat‐induced elevations of ROS might help activate proteins with Ca^2+^ channel‐related activities, such as ANNEXINS (Liao et al., 2017; Saad et al., 2020). Interestingly, the levels of ROS‐inducible Ca^2+^ channels can be tightly regulated by TFs (Liao et al., 2017), suggesting a mechanism that helps determine the optimal level of the HSR. Meanwhile, calcium and ROS play a role in activating ROS‐producing RBOHs (Kobayashi et al., 2007; Zandalinas & Mittler, 2018), revealing the cross‐talk between calcium and ROS in stress responses. Additionally, heat stress sensing also relies on the interplay between ROS and phytohormone signalling (Ang et al., 2024). While heat‐induced elevations of ROS can interfere with phytohormone production, such as ABA and auxin, phytohormones can interfere with ROS‐producing RBOH activity (Devireddy et al., 2021). Lastly, not all redox‐sensitive proteins have their function directly affected by certain heat stress conditions, as shown for HSFA8 (Giesguth et al., 2015). This observation suggests that these proteins may not be able to induce the HSR or have their functions tied to a heat stress dose‐dependent increase in ROS accumulation.

In the case of the indirect effect, key redox‐regulatory enzymes are modified by heat stress‐induced RMS, subsequently altering downstream stress response pathways in either a gain‐of‐function or loss‐of‐function signalling context. Plant thiol peroxidases, such as peroxiredoxins (PRXs) and glutathione peroxidases (GPXs), serve as important antioxidant proteins that can also be involved in regulating the expression of HSR genes, among other stress‐related functions (Vogelsang & Dietz, 2022). For instance, besides helping control intracellular peroxide levels, the Arabidopsis plastid 2‐CYS PEROXIREDOXIN A (2CPA) can contribute to a sophisticated heat stress sensing mechanism with multiple components. This mechanism begins with the heat stress‐induced production of RMS, specifically 12‐oxo‐phytodienoic acid (OPDA), which leads to the inactivation of 2CPA and ultimately culminates in the retrograde regulation of the HSR (Liu et al., 2020; Vogelsang & Dietz, 2022). Additionally, dehydrogenases, primarily involved in metabolism, can also be key regulators of the cellular redox potential, oxidative damage repair, and other functions (Jethva et al., 2023; Kültz, 2005). One particular example is the cytosolic GLYCERALDEHYDE‐3‐PHOSPHATE DEHYDROGENASE (GAPC), which, when modified by heat stress‐induced ROS, undergoes nuclear translocation and association with the TF NUCLEAR FACTOR Y SUBUNIT C10 (NF‐YC10) to trigger the HSR (Kim et al., 2020).

In summary, plants can sense heat stress when signalling proteins and key redox regulatory proteins are modified by stress‐induced RMS, which subsequently triggers the HSR. Some of these sensors, such as the HSFA4A and the GAPC, have been identified, and their mode of action revealed (Andrási et al., 2020; Kim et al., 2020). Many other cellular proteins can undergo ROS‐driven oxidative modifications upon heat stress (Babbar et al., 2021), but the impact of these modifications on the HSR remains largely unknown. Moreover, while numerous RMS‐driven protein modifications have been documented in response to various abiotic stresses, their implications under heat stress remain unexplored. For example, nitric oxide, a stress‐related RMS, has been shown to enhance the activity of Arabidopsis PROTEIN ARGININE METHYLTRANSFERASE 5 (PRMT5) (Hu et al., 2017). Stress‐activated PRMT5 then methylates RNA‐binding proteins, such as SM‐LIKE PROTEIN 4, affecting the splicing of stress‐related genes (Agrofoglio et al., 2024). The extent to which this regulatory pathway contributes to the HSR remains an open question. Therefore, additional studies on the oxidative regulation of proteins and small molecules may identify important mechanisms by which plants sense and respond to heat stress, further validating the central role of ROS in orchestrating plant thermotolerance.

### Biological membrane damage sensors

Biological membranes are highly ordered, protein‐containing phospholipid bilayers that form a continuous barrier around cells and membrane‐bound organelles. They play a fundamental role in regulating the exchange of most molecules to and from the compartments they enclose, which is essential for maintaining intricate metabolic pathways and storing crucial information. Stress‐induced alterations in membrane components – both lipids and proteins – can negatively impact their function, ultimately dysregulating cellular systems. While stressful conditions often affect membrane properties such as fluidity, integrity, stability, and permeability, the extent of stress‐induced membrane damage is influenced by these same properties (Saidi et al., 2010; Scholz et al., 2024). The relationship between membrane properties and their heat‐induced damage will be mentioned below.

Heat stress can damage membranes in several ways. First, an increase in temperature directly increases the molecular motion of lipids, leading to greater fluidity in biomembranes. The ratio of saturated and unsaturated fatty acid chains is directly correlated with membrane fluidity; a higher degree of unsaturation in fatty acid chains results in increased fluidity. Interestingly, fatty acid desaturases — enzymes that convert saturated fatty acids into (poly)unsaturated fatty acids — become unstable at elevated temperatures (Niu & Xiang, 2018). This instability could play a role in diminishing membrane fluidity during heat stress. The higher molecular motion also increases transbilayer translocations, affecting lipid asymmetry. Solid evidence linking lipid translocation to heat stress sensing in plants remains elusive. Nevertheless, changes in membrane fluidity and lipid asymmetry affect key interactions among lipids and proteins, which are necessary for maintaining membrane stability. As a consequence, membrane microdomains are disturbed and the membrane integrity is disrupted. One of the best examples of this disruption occurs in thylakoid membranes. Heat stress‐induced destabilisation of these membranes uncouples the elements of the electron transport chains, resulting in electron leakage to molecular oxygen and incomplete water oxidation. Crucially, excessive amounts of ROS are generated (Pospíšil, 2016), resulting in post‐translational modifications of membrane‐bound proteins and increased lipid peroxidation, further contributing to the loss of membrane integrity (Farmer & Mueller, 2013; Hendrix et al., 2023). Moreover, loss of membrane integrity can lead to increased membrane leakiness, including a loss of the proton gradient of thylakoid membranes, as well as increased water and solute permeability.

Surveillance systems that detect and respond to membrane damage are indispensable for sensing heat stress. Protein sensors play a key role in this process and can be divided into two groups. The first group consists of proteins that become functional in response to the increased membrane fluidity and redox imbalance induced by heat. For instance, CYCLIC NUCLEOTIDE GATED CALCIUM (CNGC) channels are mechanosensitive ion channels involved in developmental pathways and responses to environmental changes, including temperature fluctuations (Jarratt‐Barnham et al., 2021). Small or large alterations in the plasma membrane fluidity, such as those caused by heat stress, activate specific CNGCs, which in turn results in a calcium influx into the cytosol — an important signal for triggering the HSR (Cui et al., 2020; Finka & Goloubinoff, 2014; Jarratt‐Barnham et al., 2021; Kerbler & Wigge, 2023). In the mitochondrial membrane, heat‐induced redox imbalance, high Ca^2+^ concentrations and other unknown heat stress‐induced factors lead to the formation of permeability transition pores, the protein identity of which are not fully known but might involve adenine nucleotide translocators and the ATP synthase (Ocampo‐Hernández et al., 2022). Through these pores, CYTOCHROME C (CYTC) is released from the mitochondria and enters the cytosol, from where it performs secondary functions related to the HSR, such as regulating cell death and acting as a retrograde signal to the nucleus (Selinski et al., 2024). Therefore, proteins involved with the formation of the mitochondrial permeability transition pores are strong candidates for heat stress sensors. CYTC, on the other hand, is likely a secondary transducer of high membrane permeability induced by heat stress. Recently, Zhang et al. (2022) identified a plasma membrane–localised E3 ligase, Thermo‐tolerance 3.1 (TT3.1), which translocates to endosomes upon heat stress. This translocation initiates a cascade of events that protects thylakoids from heat stress. The authors suggest that TT3.1 may function as a thermosensor; however, further research is needed to confirm if TT3.1 is a temperature sensor, a primary sensor of heat‐induced membrane damage, or a secondary transducer of heat stress information.

Another important example of heat stress sensors of membrane damage is plant phospholipases (PLases). Heat‐induced changes in membrane properties, often accompanied by ROS‐mediated oxidation, allow key PLases to hydrolyse phospholipids into smaller components. Some of these components, such as phosphatidic acid (PA) and inositol‐1,4,5‐trisphosphate (IP_3_) can influence signalling networks (Ali et al., 2022; Farmer & Mueller, 2013; Song et al., 2020). The heat stress‐induced activity of several plant PLases, such as PLDδ and PLC5 has been shown to impact microtubule dynamic organisation, stomatal movement, and overall plant thermotolerance (Annum et al., 2023; Kim et al., 2022; Song et al., 2020). Notably, Kim et al. (2022) presented a fascinating finding suggesting that heat‐induced PA levels, catalysed by PLDδ, help ROS‐oxidised GAPC in its nuclear translocation. This discovery connects two pivotal mechanisms for sensing heat stress: membrane damage detection and redox imbalance sensing.

In the second group, heat stress sensing occurs via lipid peroxidation, which can significantly damage membranes and cells because it often results in lipid fragmentation and downstream generation of toxic products. Most heat stress‐induced lipid peroxidation reactions are initiated by ROS, whereupon highly reactive, lipid‐derived chemical species can be further processed enzymatically or non‐enzymatically into new components, such as reactive electrophile species (RES) and reactive carbonyl species (RCS) (Farmer & Mueller, 2013; Yamauchi et al., 2008). As mentioned in the previous section, the heat‐induced increase in RMS levels can propagate through the cell and covalently modify proteins, ultimately triggering the HSR.

Thus far, it is evident that cells can sense heat stress‐induced changes in membrane properties and lipid peroxidation, resulting in the activation of the HSR. However, the complete mechanisms of membrane‐associated stress sensing and signalling are still not fully understood. This challenge is attributed to the technical limitations in analysing the intricate nature of membrane systems, which encompass a vast array of membrane components characterised by diverse localisations, interactions, and functions (Niu & Xiang, 2018). Consequently, many questions remain unresolved and need further investigation to understand the precise role of biological membranes in plant heat stress sensing and signalling.

### Protein damage sensors

Protein function primarily depends on its chemical composition and three‐dimensional structure, both of which are affected by heat stress (Lyu et al., 2023; Volkening et al., 2019). This impact generally occurs in the form of oxidative damage or disruption of non‐covalent interactions, such as hydrogen bonds and van der Waals forces. As a consequence, many proteins begin to unfold. Unfolded proteins not only lose their functionality but also expose hydrophobic amino acid residues, which can lead to deleterious protein aggregation (Richter et al., 2010). Interestingly, not all protein aggregation is deleterious. Heat stress‐induced aggregation of key proteins containing intrinsically disordered regions can lead to their separation from the surrounding liquid phase, forming more condensed, membrane‐less droplets in a process known as liquid–liquid phase separation (LLPS) (Liu et al., 2023). In the cytoplasm, these liquid‐like droplets can lead to the formation of heat stress‐related biomolecular condensates, namely, processing bodies and stress granules (Dignon et al., 2019; Liu et al., 2023). They have a crucial role in the HSR and will be mentioned in the next section due to their involvement with RNA molecules. It is important to note, however, that not all proteins capable of LLPS act as heat stress sensors; some function as temperature sensors, such as EARLY‐FLOWERING‐3 (ELF3) and PHYTOCHROME‐INTERACTING FACTOR 7 (PIF7) (Chen et al., 2022; Jung et al., 2020). Although they play important roles in temperature responses like thermomorphogenesis and thermotolerance, they are not central to the activation of the HSR. Recently, Bohn et al. (2024) demonstrated that the thermosensor THERMO‐WITH ABA‐RESPONSE 1 (TWA1) becomes active through conformational changes in response to elevated temperatures, facilitating the early HSR. However, the integration of TWA1 with the early HSR remains unclear. For example, is TWA1's signalling pathway dependent on heat‐induced oxidative stress, or do its target genes play a role in the initial repression of the HSR? Further research should explore the mechanisms by which thermosensors could affect the HSR.

Given the profound impact of temperature on proteins, plants have three major mechanisms of thermotolerance that counteract heat stress‐induced protein damage: (i) repairing or preventing protein oxidative damage with redox‐regulatory stress proteins; (ii) stabilising or refolding structurally damaged proteins with the help of chaperones; and (iii) removing damaged proteins via proteolysis. Sensors of protein oxidative damage were already discussed in the section on ‘sensors of redox imbalance.’ Below, I address sensors that detect unfolded proteins during heat stress.

Plants have an unfolded protein response pathway for the ER (UPR) and another one for the cytosol (CPR) (Mittler et al., 2012; Ryo Kataoka & Suzuki, 2017). Not surprisingly, heat stress can activate both pathways with the help of sensors of protein damage. For example, under unstressed conditions, the ER‐localised chaperone BINDING IMMUNOGLOBULIN PROTEIN (BIP) binds to TF BASIC‐LEUCINE ZIPPER 28 (BZIP28) and co‐chaperone BCL‐2‐ASSOCIATED ATHANOGENE 7 (BAG7), retaining them in the ER. As heat‐damaged proteins accumulate inside the ER, they compete with BZIP28 and BAG7 for binding to BIP, ultimately resulting in BIP dissociating from both proteins. This process has two important outcomes: first, BIP binds to unfolded proteins, preventing their aggregation in the ER; second, BZIP28 and BAG7 are released, allowing them to separately begin their journey towards the nucleus, where they help activate the expression of UPR‐related genes (Li et al., 2017; Srivastava et al., 2013). Similarly, the CPR is triggered by the accumulation of misfolded proteins in the cytosol, which outcompete heat shock factor (HSF) proteins for binding with HSPs, such as HSP70 and HSP90 (Andrási et al., 2020; Li et al., 2010; Sugio et al., 2009). This competition not only enables HSP70/HSP90 complexes to bind to unfolded proteins and prevent their aggregation but also releases HSFA1‐type factors, promoting further expression of genes related to the CPR (Andrási et al., 2020; Wang et al., 2004). In summary, chaperones have an important role in sensing heat stress‐induced protein damage, upon which they release key TFs responsible for activating two subcomponents of the HSR, the UPR and CPR pathways.

### DNA and RNA damage sensors

The integrity of the two fundamental nucleic acid polymers, DNA and RNA, can be affected by heat stress, which serves as a signal to initiate the HSR. Much of the research investigating the sensing mechanisms of stress‐induced damage to nucleic acids has focused on DNA damage sensing in animal systems, particularly in cancer research (Nisa et al., 2019). Nonetheless, several lines of evidence indicate that heat stress also affects the integrity of both DNA and RNA in plants. Key mechanisms involved in sensing this macromolecular damage are being uncovered.

Various mechanisms through which heat stress induces changes in plant genome integrity have been described. DNA damage, such as ROS‐derived strand breaks and nucleotide modifications, can increase upon heat stress, often leading to cell cycle arrest and PCD (Han et al., 2021; Nisa et al., 2019; Szurman‐Zubrzycka et al., 2023; Vanderauwera et al., 2011). Additionally, heat stress interferes with the formation of double‐strand breaks during meiosis, altering crossover patterns and potentially affecting fertility (Modliszewski et al., 2018; Ning et al., 2021; Zhao et al., 2023). It is also associated with modifications in chromatin structure, such as changes in DNA methylation patterns, siRNA levels, and nucleosome occupancy, all of which impact the HSR (Cai et al., 2024; Pecinka et al., 2010). The genomic instability resulting from heat stress can be further influenced by transposable elements (TEs) (Fan et al., 2023; Niu et al., 2024). For instance, TE‐mediated repression of the *Hsf2Ad* rice gene during heat stress conditions has been associated with decreased thermotolerance (Wu et al., 2022). The molecular mechanisms by which plants attempt to cope with heat‐induced changes to genome integrity are increasingly being uncovered, confirming their link to the induction of thermotolerance (Han et al., 2021).

Plant cells have different mechanisms that try to minimise heat stress‐induced DNA damage. First, ROS‐scavenging enzymes outside the nuclei can form a protective barrier that reduces ROS levels, preventing them from reaching the nuclei. For example, single knockout mutants of ROS‐scavenging enzymes ASCORBATE PEROXIDASE 1 (APX1) and CATALASE 2 (CAT2) are unable to prevent H₂O₂‐induced DNA damage, resulting in a lower thermotolerance phenotype (Vanderauwera et al., 2011). Additionally, plants possess highly conserved DNA repair mechanisms that help protect their genomes against heat stress, thereby increasing thermotolerance (Han et al., 2021). One example is the Arabidopsis TELOMERIC PATHWAYS IN ASSOCIATION WITH STN1 (TEN‐1), which exhibits a heat‐induced chaperone activity that maintains telomeric DNA integrity under heat stress (Lee et al., 2016). Furthermore, Han et al. (2020) demonstrated that HIGH EXPRESSION OF OSMOTICALLY RESPONSIVE GENES 1 (HOS1) induces thermotolerance by activating DNA repair components. However, despite these various mechanisms that try to minimise DNA damage during heat stress, a high intensity of heat stress can lead to redox imbalance in the nucleus and the inhibition of most of the DNA repair mechanisms, further compromising DNA integrity, which can even culminate in PCD (Han et al., 2021).

Most of the proteins involved in DNA damage sensing are highly conserved among eukaryotes (Kültz, 2005) and have also been identified in plants (Yoshiyama et al., 2013). These include homologues of the MutS family (AbdelGawwad et al., 2019; Adé et al., 2001; Lario et al., 2011; Van Marcke & Angenon, 2013; Zhao et al., 2020), the MutL family (Alou et al., 2004; Colas et al., 2016; Dion et al., 2007; Mao et al., 2021; Xin et al., 2021), and proteins of the MRE11–RAD50–NBS1 (MRN) complex (Bundock & Hooykaas, 2002; Das et al., 2022; Gallego et al., 2001; Nair et al., 2021). However, the specific roles of these plant proteins in sensing heat stress‐induced DNA damage and regulating thermotolerance are still poorly understood. In Arabidopsis, heat stress mostly downregulates the expression of *MSH2* and *MSH6* (Lario et al., 2011), while depletion of the plastid protein AtMSH1 results in increased thermotolerance (Virdi et al., 2016). Phillips et al. (2015) found that, under higher temperatures, HvMLH3 foci are repositioned during barley male meiosis, a phenomenon linked with altered crossover patterns. These results suggest that DNA‐damage‐sensing proteins are involved in plant thermotolerance mechanisms; however, further analyses are needed to confirm their involvement in sensing heat stress, which is no easy task. Distinguishing between primary sensors and secondary transducers of DNA damage is challenging because of the complex regulatory network of proteins involved in DNA damage sensing (Kültz, 2005). For example, the MRN complex recruits ATAXIA‐TELANGIECTASIA MUTATED (ATM) to sites of DNA double‐strand breaks (DSBs), thereby activating ATM's kinase activity. ATM then rapidly phosphorylates several target proteins, including nearby variant forms of the histone protein H2A, creating a crucial epigenetic signal that recruits the DNA damage response (Shibata & Jeggo, 2021; Yoshiyama et al., 2013). Zhao et al. (2023) have shown that ATM‐mediated DSB repair is required for maintaining meiotic chromosome integrity in plants under heat stress. This process occurs downstream of the MRN complex. Therefore, their work suggests that MRN and ATM are involved in sensing and transducing the signal of heat stress‐related DNA damage in plants, respectively. Overall, it is clear that DNA damage sensors are well conserved across eukaryotes, and understanding how these proteins function during heat stress will improve our knowledge of heat‐induced DNA damage sensing.

Heat stress‐induced RNA damage has also been identified in plants and can occur in distinct ways. For example, Merret et al. (2015) have shown that in Arabidopsis, heat can trigger 5′‐ribosome pausing, leading to the decay of translating mRNAs mostly coding for heat‐sensitive proteins. This phenomenon is partly explained by an HSP70‐dependent sensing activity and an increased influx of mRNPs into processing bodies (Merret et al., 2015; Weber et al., 2008). Additionally, acute heat stress can more easily melt A:U‐rich secondary structures of mRNA compared with G:C‐rich ones. Consequently, A:U‐rich UTRs become easier substrates for degradation by exonucleases (Su et al., 2018). This phenomenon allows for a quick reorganisation of the reservoir of translatable mRNAs upon acute heat stress, raising the question of whether it could help select HSR‐related transcripts with G:C‐rich UTRs. In this case, the UTRs of RNAs could function as heat stress sensors, becoming potential candidates for genetic engineering for increased thermotolerance (Su et al., 2018).

While specific transcripts can be more easily degraded upon a stress signal, others can have increased protection from heat‐induced degradation, resulting in increased thermotolerance. This phenomenon involves RNA binding proteins (RBPs), such as OLIGOURIDYLATE BINDING PROTEIN 1 (UBP1), that have stress‐induced LLPS capabilities associated with the stability of their target transcripts (Nguyen et al., 2016; Weber et al., 2008; Yan et al., 2022). These RBPs may be acting as sensors or secondary transducers because of their ability to finely prevent heat stress‐induced RNA degradation. For example, Tong et al. (2022) observed that the RNA‐binding protein (RBP) ACETYLATION LOWERS BINDING AFFINITY (ALBA) binds to and stabilises HSF mRNAs upon heat stress. The authors observed that ALBA proteins phase separate into cytoplasmic granules upon heat stress, known as stress granules (Weber et al., 2008), where they recruit and stabilise several HSR transcripts. As expected, these transcripts are degraded in thermosensitive *alba* mutants undergoing a heat stress treatment, corroborating the hypothesis that ALBA proteins stabilise HSR transcripts and increase thermotolerance. More recently, Wu et al. (2024) discovered that branched circular RNAs, known as lariRNAs, become less degraded under heat stress. This heat‐induced accumulation of lariRNAs occurs because the enzyme responsible for their turnover, RNA DEBRANCHING ENZYME 1 (DBR1), is sequestered from the nucleus to cytoplasmic stress granules by SICKLE, the direct interactor of DBR1 with LLPS capabilities. With reduced DBR1 in the nucleus, lariRNAs accumulate, leading to increased transcription of their heat stress‐related parental genes and ultimately contributing to enhanced thermotolerance (Wu et al., 2024). It would be interesting to explore whether other proteins with LLPS capability, such as RBP DROUGHT RESISTANCE GENE 9 (Wang et al., 2024) and RNA‐BINDING GLYCINE‐RICH GROUP D 2/4 (Zhu et al., 2022), can regulate gene expression upon heat stress. Lastly, proteins that mediate the assembly of processing bodies and stress granules, both involved in heat stress‐related mRNA processing, are valuable candidate proteins that may sense heat stress and help trigger the HSR (Zhu et al., 2022).

## SYSTEMS VIEW ON HEAT STRESS SENSING

The potential heat stress sensors mentioned here make up a useful list of genes involved in triggering the HSR. Additional studies are required to fully identify and characterise all the key components responsible for activating this response. However, studying heat stress sensing remains challenging due to its complexity, and adopting a systems view could offer a crucial way forward. In this approach, besides identifying the sensory components, it is also important to consider their interaction with other elements, and the context in which they operate. For example, two or more types of heat stress sensors discussed in this review could act simultaneously under heat stress. Their signalling cascade can interact, which usually gives rise to emergent properties, that is, novel functions that arise from their interaction. For instance, ROS produced during heat stress can interfere with other signalling pathways, such as the DNA damage response, thereby enhancing the HSR (Vanderauwera et al., 2011). Another example, mentioned previously, is the work by Kim et al. (2022). They presented an interesting finding connecting heat stress‐induced membrane damage and redox imbalance in mediating thermotolerance. However, it is important to note that the contribution of the different heat stress signalling cascades likely varies with the plant's developmental stage, time of day, and season (Dickinson et al., 2018; Grossman, 2023; Huang et al., 2012), as heat‐induced macromolecular damage is influenced by the tissue's physiological state. For instance, tissues at later developmental stages may prioritise maintaining membrane stability (Nijabat et al., 2020) and protecting photosystems (Pshybytko et al., 2023) compared with younger tissues. Thus, a comprehensive understanding of heat stress sensing will require not only the identification of individual sensors but also an integrated approach that considers the broader signalling networks they influence, along with the specific conditions in which they act.

Currently, there is no evidence of a single element within the plant cell serving as a definitive control centre for the HSR, capable of integrating inputs from all heat stress sensors. Signalling hubs, such as HSFs and some NAC‐type transcription factors, may function as integrators by responding to upstream signals and coordinating the downstream HSR through the nucleus (Bakery et al., 2024; Takahashi et al., 2019). Within the nucleus, multiple signalling inputs are processed simultaneously, enabling it to orchestrate complex gene regulatory networks efficiently (Kan et al., 2023). This capacity for parallel processing allows the nucleus to integrate signals from various heat stress sensors and thermosensors to manage both the HSR and CHR under heat stress conditions. While this may already seem intricate, the integration of these signals is far more complex under natural conditions.

In natural conditions, stressors rarely act in isolation, and plants have evolved a complex regulatory network that senses and responds to multiple stresses simultaneously (Zandalinas et al., 2024). While some heat stress sensors are specific to heat, others can respond to multiple stresses, either in conjunction with or independently of other stress factors, making it challenging to understand heat stress sensing fully when studied in isolation. For example, there are different responses observed under combined heat stress and drought stress, as well as heat stress and high light stress that are absent under a single stress (Balfagón et al., 2019; Sato et al., 2024; Xu et al., 2023). Understanding how cells detect the different stress‐related stimuli and translate this information into an appropriate combined‐stress response, could enable the development of crops with greater tolerance to major abiotic stresses that often occur simultaneously in many of the areas used for crop production. Another example of this complexity is the relationship of heat stress with organismal interactions. Adams et al. (2022) observed a negative shift in ecological interactions between organisms, including plants, as heat stress increases, indicating disruptions or reductions in mutually beneficial relationships or other ecological dynamics. Could plant heat stress sensors be involved in this shift? Perhaps, systems biology and its network modelling approaches can be considered in these challenging studies for their ability to extract useful information from complex biological systems (Kültz, 2020). In parallel, new tools involving nanotechnology are continuously being developed to study stress sensing. Recently, Zhang et al. (2024) developed a polymeric nanocarrier‐based platform capable of delivering redox‐sensitive GFP into plant cells, opening new ways for studying protein‐based sensing mechanisms and their applications. Ang et al. (2024) have developed nanosensors able to monitor stress‐induced H₂O₂ and salicylic acid in plants subjected to distinct stress treatments, including heat stress, demonstrating that sensor multiplexing can be used to study stress signalling mechanisms in plants.

## CONCLUSIONS

This Focused Review examines the major mechanisms by which plants sense heat stress (Box 1). These and future findings in the field could lead towards the much‐needed practical applications in agriculture. Agronomic management practices aimed at protecting crops from heat stress have mostly focused on escape and resistance strategies, such as adjusting planting dates and supplemental irrigation, respectively (Berger et al., 2016; Khan et al., 2019). However, these strategies are reaching their maximal effectiveness, and further pursuit in this direction might negatively impact crop production (Osmond et al., 1987). In contrast, considerable potential remains for improved productivity when these practices are complemented by thermotolerance strategies (Khan et al., 2019; Osmond et al., 1987). Here, I have discussed that a primary aspect of thermotolerance is heat stress sensing and studies focused on the initial components responsible for activating the HSR have the potential to significantly impact agriculture. For example, cytological mapping of HvMLH3 foci has shown that crossovers can shift to more proximal regions of the chromosomes simply by elevating the growth temperature, a finding that could hold great value in barley breeding programs (Phillips et al., 2015). The use of nanomaterials with ROS‐generating or ROS‐scavenging properties has been used as priming agents to enhance plant tolerance to multiple abiotic stresses, including heat stress, though it is necessary to further explore the mechanisms by which these nanomaterials affect stress sensing and responses (Bao et al., 2024; Chen et al., 2023). In summary, advancing our knowledge of how heat stress, and its combination with other stresses, are sensed at the molecular level (Box 2) offers a promising avenue for enhancing crop thermotolerance – knowledge that can further aid in protecting plant biodiversity and even help explain species distribution and the evolutionary processes driven by heat stress‐related selection pressure.

**Summary**

- At the molecular level, heat stress is often defined as any particular rise in the optimum growth temperature that pushes the physiological status of a cell beyond its homeostatic range, leading to a system dysregulation.
- Thermotolerance is one of the three strategies related to plant adaptation to heat stress and is perhaps the most dependent on the perception of heat stress information.
- Although the heat stress response (HSR) and the cellular homeostasis response are linked and contain common elements, they are different types of responses, with many sensing‐ and signalling‐specific pathways.
- If a plant cell is subjected to a temperature increase that exceeds its optimum range, many cellular systems become impaired and dysregulated, resulting in particular redox imbalances and certain types of macromolecular deformation/damage, namely on lipid membrane, protein, and nucleic acids.
- Heat stress‐specific redox imbalance and macromolecular damage are detected by heat stress sensors, upon which they would immediately promote and maintain the appropriate level of the HSR.

**Open Questions**

- What are the cellular signatures of reactive molecular species and redox‐regulatory proteins that are able to trigger the HSR?
- What specific roles do proteins involved in DNA damage sensing play in detecting heat stress‐induced DNA damage and driving thermotolerance?
- Can proteins with liquid–liquid phase separation capabilities and proteins that mediate the assembly of processing bodies and stress granules function as heat stress sensors?
- How do the different heat stress sensors integrate to trigger the optimal level of the HSR?
- How do cells detect multiple stress‐related stimuli and translate this information into an appropriate combined stress response?

## Conflict of Interest Statement

The author declares no conflict of interest.

## Data Availability

Data sharing not applicable to this article as no datasets were generated or analysed during the current study.
